# Predictors of sustained physical activity: behaviour, bodily health, and the living environment

**DOI:** 10.3389/fphys.2023.1213075

**Published:** 2024-01-08

**Authors:** Delia Elena Diaconașu, Iulian Stoleriu, Ioana Andreea Câmpanu, Ana-Maria Andrei, Ștefan Boncu, Cezar Honceriu, Veronica Mocanu, Georgiana Juravle

**Affiliations:** ^1^ Sensorimotor Dynamics Laboratory, Faculty of Psychology and Educational Sciences, Alexandru Ioan Cuza University, Iasi, Romania; ^2^ Department of Social Sciences and Humanities, Institute of Interdisciplinary Research, Alexandru Ioan Cuza University, Iasi, Romania; ^3^ Faculty of Mathematics, Alexandru Ioan Cuza University, Iasi, Romania; ^4^ Faculty of Educational Sciences, Stefan cel Mare University, Suceava, Romania; ^5^ Faculty of Psychology and Educational Sciences, Alexandru Ioan Cuza University, Iasi, Romania; ^6^ Faculty of Physical Education and Sports, Alexandru Ioan Cuza University, Iasi, Romania; ^7^ Department of Morpho-Functional Sciences 2—Pathophysiology, Grigore T. Popa Medical University of Iasi, Iasi, Romania

**Keywords:** walking, environment, health, risk for cardiovascular disease (CVD), BMI, blood cholesterol, physical activity, Artificial Neural Networks (ANNs)

## Abstract

This study examined the determinants of sustained physical activity. Eighty-four participants undertook a 7-weeks walking regime (i.e., a 1-h biometrically-monitored walk, at least 5 days/week), with bioelectrical impedance (BIA) and total cholesterol capillary blood measurements performed before and after programme. To investigate behavioural habit formation, 7 weeks after walking termination, all participants were interviewed and (health) re-tested. Data were modelled with an artificial neural network (ANN) cascading algorithm. Our results highlight the successful prediction of continued physical activity by considering one’s physical fitness state, the environmental living context, and risk for cardiovascular disease. Importantly, those artificial neural network models also taking body mass index (BMI) and blood cholesterol as predictors excel at predicting walking continuation (i.e., predictions with 93% predictability). These results are first to highlight the type and importance of available physiological drivers in maintaining a sustained physical activity regime such as walking. They are discussed within the framework of habit formation and the nowadays health and/or wellbeing focus.

## Introduction

A physically-active regime significantly impacts health and longevity. People who regularly exercise have better immunity and a grander resistance in the face of disease (Macciochi, 2020). It has been demonstrated that purposefully moving our body promotes health in various ways. For example, muscle mass may be gained, and the heart muscle tonus and functionality are maintained, together with a healthy blood pressure and cholesterol level (O’Mara, 2019). Conveniently, health-promoting exercise needs not necessarily be organized around a group, or even be time-, and/or resource-consuming: Exercise can be as simple and as available as an everyday walk. Walking regularly promotes health, as it concomitantly activates the cardiovascular, respiratory and locomotor systems, with specific resultant physiological effects, including a better oxygenation of the body and an enhanced vital capacity. Walking has been proposed to be closest to the perfect physical exercise (Morris and Hardman, 1997). It is performed both unintentionally, within the scope of daily activities, or intentionally, such as the recreative walking through a park or garden (Kelly et al., 2017). Since walking is free, low-income groups are likelier to engage in regular moderate walking (Blacklock et al., 2007), whereas the pay-for-use-facility physical activity tends to be encountered in individuals with higher income (Taylor, Floyd, Whitt-Glover and Brooks, 2007).

There is unanimous agreement that walking offers a natural, widely accepted, low injury risk, social functioning and environmentally friendly approach to physical activity. Walking at a self-selected pace is of moderate intensity for most adults (Murtagh et al., 2015). Further, daily walking has been demonstrated to enhance cognitive function (Zheng et al., 2016), promote a better sleep (Wang and Boros, 2021), sustain weight loss when practiced over longer durations (Hanson and Jones, 2015), lower the incidence of CVD, obesity, and Alzheimer’s disease (Reiner et al., 2013), as well as to regulate blood circulation for those cases where one remains seated for longer periods of time (Carter et al., 2018). Defining a healthy walking workout is conditioned by age, gender, and the general health state. For example, take the older population, for whom walking can constitute a significant moderator for social engagement (Carrapatoso et al., 2017). Physical effort volume and intensity, but even its complexity, needs to be adjusted such that the bodily adaptation is optimally achieved.

In the context of walking, we can nowadays easily access biometric information via smart wearable devices. Diverse metrics are offered as indicators of health and appropriate health behaviour, across a variety of fields, such as sleep (Wen et al., 2022), stress (Pakhomov et al., 2020), chronic disease management and sports or preventive medicine (Casselman et al., 2017; Vogel et al., 2017), as well as, in what regards children safety or military support (Casselman et al., 2017). Furthermore, while measurements of health-related indices exist in connection to physical activity and exercising, as well as their beneficial influence in various pathologies (Bowler et al., 2010; Berman et al., 2012; Marselle et al., 2013; Williams and Thompson, 2013), the specific type of physical activity assessment, but also its optimal time-span, needs to be considered.

For example, significant general contributions of exercising to health have been reported (del Pozo et al., 2022a; del Pozo et al., 2022b) see also recent research reports on participants re-tested several years from the first evaluation of physical activity and health status (Casselman et al., 2017; Vogel et al., 2017). However, the majority of previous research has concentrated on the *self-reported* physical activity intensity and frequency of it, or had before/after designs of a single bout of a given motor behaviour evaluation (Hartig et al., 2003; Roe and Aspinall, 2011). Nevertheless, longitudinal 6-month monitoring studies of walking do exist, e.g., in the form of the British *Walk to wellbeing* study, with participants taking walks either in the city or in nature (Marselle et al., 2013). For longitudinal studies, the most evident barrier and risk in conducting a real-life monitoring study is participant engagement and, consequently, the scale of study participants drop-outs. If the study participants recruiting can be conveniently handled through a student population motivated through the offering of study credits (i.e., such is the case of the present study approach), the intended sustained motivation for the participation in the study needs the support of various planned strategies (e.g., amongst those that were used in the present study: initiating the study in January, simultaneously with any potential New Years’ resolutions; a well-trained team of research assistants, closely monitoring the adherence to the required walking regime).

Location appears to affect walking. Environmental psychologists have demonstrated the benefits of natural landscapes for physical and mental health, a positive mood and a significantly reduced stress, improved social and material wellbeing as the main benefits for being in nature and having contact with nature, with such effects found to be present across cultures (Kaplan and Kaplan, 1989). One of the earliest empirical tests on the healing qualities of contact with nature contrasted two types of views from a hospital room, as reflected in patients’ recovery period: Patients in rooms with windows facing a park registered less days of hospitalization compared to those positioned in salons facing the inner courtyard, had fewer post-operative complications and were evaluated by the medical staff as having positive affective states (Ulrich, 1984). This preference for natural habitats has highlighted the stress-reducing effect of natural, over urban environments (Ulrich, 1981; Chang and Chen, 2005; Velarde et al., 2007). Significant decreased noradrenaline and heart rates were also associated with forest walking as compared to city walking (Li et al., 2016), with walking in a park found to improve health (Barton, Hine and Pretty, 2009; Taylor, Robertson, Lightfoot, Smith and Jones, 2022; Tsao et al., 2022). For example, evidence for increased relaxation is suggested by reduced stress hormones secretion, such as cortisol, adrenaline, and dopamine, with walking in a forest, as opposed to a city environment, known to reduce heart rate and blood pressure in individuals without chronic disease (Taylor et al., 2022).

Taking into account the previous findings of walking and/or physical activity in city and natural environments, here, we evaluate and predict the continuation of a healthy habit, once a given *sustained* healthy regime has commenced. We focus on everyday naturalistic walking and investigate the health benefits of such a daily walk, as evidenced by how the environment where one lives and walks affects a person’s health. The goal of our study is to distinguish whether one is more likely to continue a common motor behaviour regime such as walking irrespective of spatial context, or rather, whether walking in specific environments (e.g., in the park) results in an overall objectively healthier body profile and propensity to transform walking into a long-term habit (Ardigo et al., 2011; Zhao et al., 2019). Health status is assessed through the body mass index (BMI), total cholesterol blood profiles, as well as the subjectively reported mood and wellbeing, together with the assessment of cardiovascular disease risk, personal physical fitness, living and environmental self-reported data. We hypothesize that the specific location of the daily walk will significantly influence participants’ drive to perform physical activity. Further, taking into consideration that most real-world systems display intricate nonlinear traits and cannot be adequately addressed using linear systems theory (Chen and Billings, 1992), we hypothesize that the propensity to continue an already started physical activity regime is given by a real-life-driven and inherently non-linear combination of a person’s behaviour, health, and the living environment.

As such, the goal of the present study is to identify those combined environmental and psychophysiological factors necessary to make walking a long-term healthy habit. For this, we utilise artificial neural network (ANNs) systems to investigate behavioural and physiological determinants of walking, so as to highlight those relevant drivers which can be easily and efficiently used to monitor the continuation of an already-started healthy physical activity programme. Considering the present complex longitudinal multi-dimensional design, ANNs are excellent models for data classification and prediction, well-known for their outstanding self-learning capabilities, adaptability, resilience to faults, inherent nonlinearity, and proficiency in mapping inputs to outputs. Importantly, they are outstanding in addressing both complex and straightforward problems across various life domains (Abiodun et al., 2018). In this context, to the best of our knowledge, this is the first longitudinal study to apply NN algorithms in order to predict the continuation of a common motor behaviour regime (e.g., sustained walking) by monitoring data from multiple sources (e.g., wearable data, objective physiological data from the assessment of BMI and blood cholesterol, as well as the assessment of self-reported cardiovascular risk, living and environmental perceptions, and fitness).

## Materials and methods

### Participants

From amongst students taking the Neuroscience undergraduate course in our university, we used convenience sampling to advertise and offer credit course for taking part in the study. Participants who expressed interest to participate were randomly distributed in one of the three experimental groups. Further, given the constraints in finding and keeping participants motivated to walk for the entire duration of the study, we also used snowball sampling, to attract friends of already-committed participants, as well as of research assistants and experimenters. Three of the study authors have taken part in the study. A total of 90 participants took part in this study. Out of these, two dropped out and four participants were excluded from the final data analysis, because of significant missing data. The remaining sample (*N* = 84) included 12 male participants and had a mean age of 25.83 years, SD = 7.84 years, age range 20–55 years. The study received ethical clearing from the Alexandru Ioan Cuza ethics committee (no. 2984/27.09.2021) and written informed consent was obtained from all participants before beginning the experiment. All participants were debriefed with respect to the study purpose at the end of the experimental session. This study conforms to the Declaration of Helsinki and to all subsequent amendments (Declaration of Helsinki, 1964, 2013).

### Materials and apparatus

The participants were allocated to one of the experimental groups: city walking–with instructions to walk within a city environment, park walking–with instructions to walk in the park/forest/garden or a green environment, and a control group–with no walking required for this group. The experimental participants were required to walk for a minimum of an hour, at least 5 days a week. The participants in the experimental groups (city and park walking) were also fitted with Mi band 5 sports bands (Anhui Huami Information Technology Co., Ltd., Anhui, China) and the corresponding application was installed on their personal smart phone. All participants were also required to fill-in a daily ecological momentary assessment (EMA) questionnaire (i.e., an experience sampling questionnaire which typically took about a minute to complete, and which was made available in the app at 6 p.m. daily and expired at 2 a.m. the following day). The daily questionnaire was delivered through the SEMA3 app (Koval et al., 2019; O’Brien et al., 2023) and included 21 questions about their current day and their affective state for the day; the data from these daily EMA assessments are reported elsewhere. These (app) requirements were monitored and addressed once per week by our research assistants, such that participants with missing data were called and reminded to comply with the walking and EMA daily questionnaires requirements.

A body composition scale was utilised for BIA assessments (RD-953, Tanita, Japan). Total cholesterol capillary blood tests were performed with a MulticareIN portable device and disposable individual tests (Biochemical Systems International SpA, Arezzo, Italy); standard measurement intervals for cholesterol (130–400 mg/dL/3.3–10.2 mmol/L) were utilised. Note that a comparable accuracy has been achieved between measurements made with such a point-of-care cholesterol testing device and traditional laboratory instruments (Rapi et al., 2009).

The data that support the findings of this study, together with the ANN Matlab code, are available from the corresponding author (GJ), upon request.

### Procedure and experimental design

**FIGURE 1:**
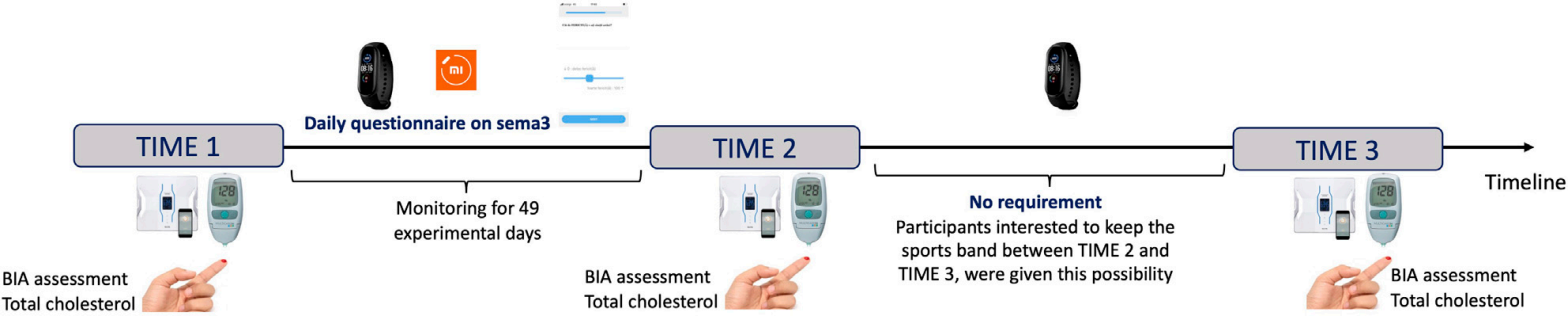
Chronological study timeline, enlisting measurements recorded for the three laboratory testing times.

The study was conducted between 11/01/2022 and 13/05/2022; see Figure 1. The experiment consisted of several measurement phases (i.e., ***Time 1***—start measurement, ***Time 2***—post-walking-regime-intervention measurement, taken at *Time 1* + 7 weeks, and ***Time 3*** –follow-up measurement, taken at *Time 1* + 14 weeks), together with a physical exercise intervention. The physical intervention requirement consisted of 6 weeks of at least 1 h/day walking, for at least 5 days/week. Note that it takes an average of 66 days for a habit to become automatic (Lally et al., 2010). In the light of the evidence-based research, the decision was taken for allowing a period of walking monitoring in this average range: That is, the initial 6 weeks of walking, together with the 6 weeks of no requirement (between *Time 2* and *Time 3*) would have given us 84 days to assess the determinants of forming a walking habit. Because the study coincided with an extended COVID-19 wave, we allowed for an extra week of walking, such that data spread would still permit to assess the forming of a habit and a participant would not be completely lost, in case a walker was kept at home with COVID during the study participation. As such, daily questionnaires and movement data were collected and analysed for 49 days between *Time 1* and *Time 2*, with a total of 98 days between walking regime start and the evaluation of its continuity, between *Time 1* and *Time 3*.

Capillary blood tests and BIA assessments were performed at each of the *Time 1*, *Time 2*, and *Time 3*. At measurement *Time 3*, all participants were also assessed on the continuation of physical activity over the previous 7 weeks, which was self-declared. For the assessment of their objective health status, at *Time 3* they also completed a cardiovascular disease risk assessment questionnaire developed in line with current guidelines for the prevention of CVD (Miller et al., 2011; Grundy et al., 2019; Pearson et al., 2021). The YES/NO questionnaire included two smoking-related questions (i.e., if currently smoking, and if they quit smoking over the past three months), together with a series of questions assessing the presence of personal/family history of various conditions including diabetes, arterial hypertension, CVD, chronic kidney disease, obesity (e.g., BMI ≥ 30), inflammatory diseases (e.g., psoriatic/rheumatoid arthritis), chronic obstructive pulmonary disease, and/or any hypertensive disorder of pregnancy. Participants also completed a 5-item questionnaire which assessed their self-reported level of physical fitness, the International Fitness Scale (IFIS, see Ortega et al., 2011; 2023). The scale includes items assessing overall physical fitness, cardiorespiratory fitness, muscular force, speed/agility, and flexibility, on a 5-point scale from 1 (poor) to 5 (very good). The calculated internal consistency for this study is acceptable, Cronbach’s *α* = .88; 95% CI [.83 .92]. Finally, participants also filled in a European-population-standardized questionnaire assessing their living environment (ALPHA, Spittaels et al., 2009). The ALPHA questionnaire consists of 49 items, organised in 9 themes, surveying the types of residences in one’s neighbourhood, the distance to various local facilities, the existing neighbourhood walking or cycle infrastructure, the maintenance of the infrastructure, the perceived neighbourhood safety and pleasantness, the existing cycling and walking network, the respondent’s home environment, as well as their workplace or study environment. The calculated internal consistency for this study is good, Cronbach’s *α* = .74; 95% CI [.69 .80].

Additionally, a selection of standardized questionnaires targeting participants’ subjective wellbeing was also filled in by participants at each of the three measurement times, online, through custom-made Google forms (i.e., the International Personality Item Pool 50, IPIP-50, Goldberg et al., 2006; Iliescu, Popa and Dimache, 2015; the Life Orientation Test revised, LOT-R; Scheier et al., 1994; the Psychological Wellbeing Scale; Ryff, 1989; the Body Appreciation Scale 2; Tylka and Wood-Barcalow, 2015; the Warwick-Edinburgh Mental Wellbeing Scale; Tennant et al., 2007). These questionnaires data are reported elsewhere.

At first measurement (*Time 1*), participants were welcomed in the lab, were given the consent form, and were required to attend a short presentation from one experimenter, outlining the setting-up of the Mi5 band, the connection with the personal smart phone, and the two apps required for participating in the study (e.g., the Mi Fit and the SEMA3 apps). At each of measurement phases (*Times 1*, *2*, and *3*), the participants were invited to step on the scale, barefooted. Their height was verbally assessed and then checked against a wall, with a linear meter; the experimenter assured the participant’s head was positioned in the Frankfort plane. Participants were weighted with their clothes on, after a series of standard questions assessing their age, gender, and their activity level (from sedentary, with little/no exercise, to moderate–occasional exercise, active–regular exercise, to athlete–more than 12 h exercise per week).

*Time 1* physiological measurements (i.e., the BMI and blood cholesterol) commenced on 11 January 2022. The in-app data collection for the 7-week walking regime app-supervised through the Mi Fit and the SEMA3 apps was initiated on 17 January 2022 and was concluded on 7 March 2022. *Time 2* physiological measurements were performed in the following week (i.e., March 7th—14th 2022). *Time 3* physiological measurements were performed between May 2nd and 13th 2022.

### Data analysis

Data pre-processing and analysis was performed in MATLAB (R2020a, MathWorks, Natick, MA, US), and implemented via the Neural Network Toolbox. Artificial Neural Networks (ANNs) permit the detection of complex *non-linear* input-output relationships between variables, affording higher performance in binary classification and pattern recognition problems, as compared to classical regression models. Importantly, the ANNs require less formal statistical training, are independent of the statistical distribution of the data and have better-discriminating power, as compared to classical regression models (see, for example, their demonstrated success over regression-type models in predicting physical activity features, Staudenmayer et al., 2009; Montoye et al., 2017). ANNs are the most common used approach to predict gait data (Caldas et al., 2020). Relatedly, with specific consideration of physical activity parameters, the superior efficiency of machine learning techniques was demonstrated in predicting exercise relapse, by using accelerometer-recorded physical activity data: Accurate robust predictions are derived especially from the number of steps and physical activity intensity (Zhou et al., 2019). In this context, our developed NN algorithm classifies, based on several variables, if one individual is likely/not to continue his/her/their already started physical activity. The ANN approach thus uses a probabilistic white-box technique, able to capture high-order relationships between sets of variables, with respect to classification. This method exhibits excellent discriminatory power, when compared with conventional methods (Abiodun et al., 2018; Yasnitsky et al., 2019; Rodríguez-Hernández et al., 2021).

At pre-processing stage 17 independent variables (i.e., age, gender, BMI, basal metabolic rate, body water, bone mass, waist circumference, fat free mass, fat mass, fat percent, muscle mass, weight, blood triglycerides, blood cholesterol, CVD risk index, CRI, environment score, ES, fitness state, FS) were considered, together with 1 dependent variable, physical activity continuation. After performing a correlation analysis on the variables, we observed that some of them were dependent on each other and were removed from the analysis (i.e., as this is typically performed, to reduce multicollinearity in the model). Taking into account the relatively reduced volume of data, we further reduced the number of independent variables to only 6 features (i.e., predictor variables) and one predicted class, which were chosen to be the ones with the most significant correlation with the response variable. This predicted class was defined as a binary variable to mirror the continuation of an already started physical activity regime, as assessed at measurement *Time 3*, with two values coded as 1 and 2. That is, code 1 indicated that a participant declared that they had less or NO continued physical activity after the experiment, whereas code 2 class indicated that the participant declared to have continued with the initial physical activity, at least as strenuous as this was performed during the experiment (i.e., a YES class). The continuation of physical activity was assessed by paper and pencil at *Time 3* measurement. The final dataset included a total of 84 observations/participants, out of which 34 observations/participants were classified in class 1 (NO), and 50 observations/participants were classified in class 2 (YES).

For the models reported in this study, we trained artificial neural networks to estimate the continuation of physical activity (***PA***), in three cascading steps, based on the study manipulated group variable (control vs. park vs. city walking), as well as the type and the typical availability of monitored data. As such, the self-reported questionnaire data is the most readily available type of monitored data, it is relatively easy to acquire, but also known to be prone to (memory) recall bias. Our first basic Model 1 was thus based on 4 predictors, including the experimental group (***EG***, control vs. city walking vs. park walking), cardiovascular risk index (***CRI***, the total score on the CVD risk questionnaire; the higher the score, the higher the risk for CVD), an environment score (***ES***, the total score calculated on the environment questionnaire, Spittaels et al., 2009) and the fitness state (***FS***, the total score on the self-assessed fitness state, Ortega et al., 2011). Model 1 was prepared as the self-report baseline for predicting the continuation of a sustained physical activity, such as walking. Models 2 and 3 include further physiological measurements: For Model 2, we included the baseline predictors utilised in the first model, to which we also added one further body composition variable (i.e., ***EG***, ***CRI***, ***ES***, ***FS***, and body mass index (***BMI***) measured at *Time 2,* following the 7-week walking regime required for the experimental group). Lastly, Model 3 was based on 6 predictors, i.e., the previously considered, ***EG***, ***CRI***, ***ES***, ***FS***, ***BMI***, together with the blood cholesterol measured at *Time 2* (***Ch***).

### Neural network architecture

**FIGURE 2:**
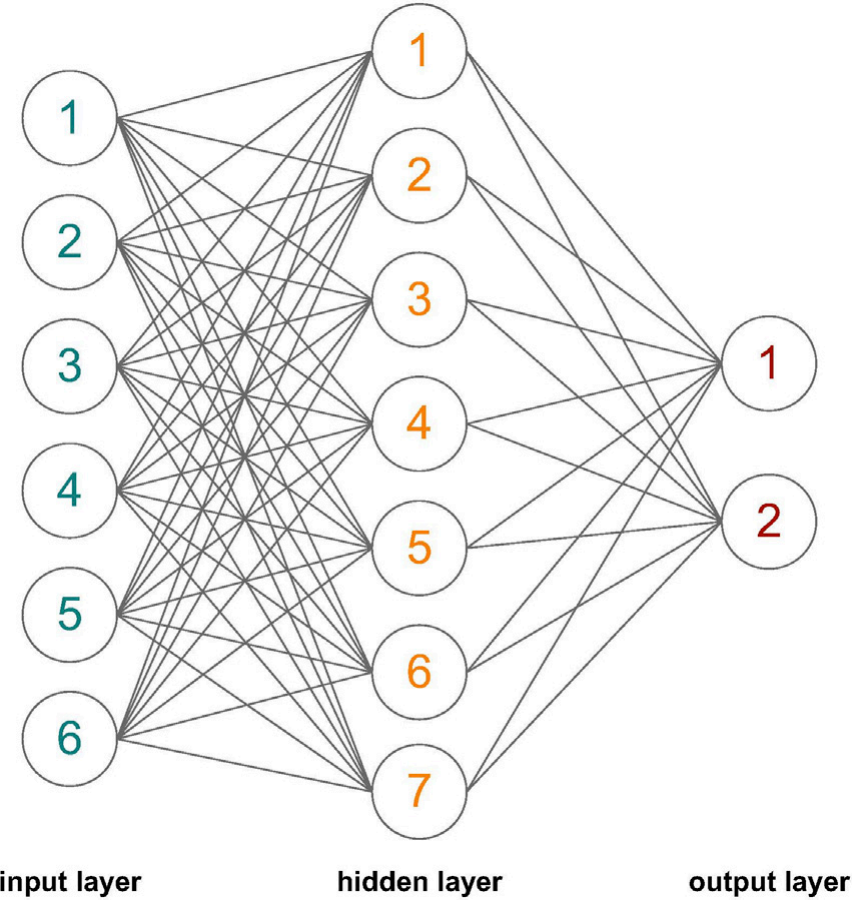
Artificial neural network architecture utilised in the present study Model 3, including one input layer with 6 neurons, one hidden layer with 7 neurons and one output layer with two neurons.

As it can be observed in Figure 2, depicting the architecture of the ANN utilised in this study for Model 3, this contains one input layer with six neurons, one hidden layer with seven neurons, and one output layer with two neurons. Depending on the model, the given inputs are experimental group (***EG***), cardiovascular disease risk index (***CRI***), environment score (***ES***), fitness state (***FS***), mean body mass index (***BMI***), and blood cholesterol (***Ch***). A trial-and-error process was utilised to determine the number of neurons in the hidden layer (i.e., 7 neurons). The activation function employed for the hidden layer was the *tansig* function:

$$f\left(x\right)=tansig\left(x\right)=\frac{2}{1+e^{-2x}}\;-1$$

For the output layer, the *softmax* function was used. The performance of the network is assessed using the MSE (mean squared error):

$$MSE=\frac{1}{n}\sum_{i=1}^{n}{\left(\hat{y_{i}}-y_{i}\right)}^{2}$$

### Neural network training method

To improve the learning performance, the dataset was randomized prior to training. The data set was then randomly divided into two separate sets, such that 80% of data values were used for the training set, and 20% for the validation set. As such, for ANN training, a back-propagation Levenberg-Marquardt algorithm with a MSE performance function was used. The Levenberg-Marquardt back-propagation ensures faster convergence speed as compared to other algorithms, while still maintaining better overall performance (Yu and Wilamowski, 2011). The MSE function is the function that is minimised during the training process within the ANNs. It represents the alteration between the original value and the predicted value of the response variable (Rajawat et al., 2022). The MSE is an important ANNs metric as it is easily calculated, penalises large errors, and it lies close to the centre of the normal distribution (Maier and Dandy, 2000).

Several networks need to be trained for the same set of data, in order to obtain a robust network performance or a solid classification for our chosen dependent variable (i.e., physical activity continuation). Therefore, we trained 50 ANNs with the architecture mentioned above, see Figure 2. Each ANN was trained starting with different initial weights and biases, and with a different division for the training and test data sets. To ensure good generalization, each ANN was retrained a further 5 times. After training, the ANN with the best performance (i.e., the lowest MSE) on the test data set was chosen to make predictions on the continuation of physical activity, ***PA***.

## Results

Averages together with standard deviations of considered bodily characteristics and total blood cholesterol, for each of the three study groups are presented in Table 1. Table 2 outlines average walking data for the experimental groups of city and park walking. Descriptive data for the total scores regarding the cardiovascular disease risk index (Miller et al., 2011; Grundy et al., 2019; Pearson et al., 2021), the environment score (Spittaels et al., 2009), and fitness state (Ortega et al., 2011) for each of the three study groups are reported in Table 3. Note that with an overall average BMI of 23.55 kg/m^2^ across the study groups and measurement times, our sample is positioned in the upper range of the ideal BMI, corresponding to normal weight (18.5–24.9).

**TABLE 1:** Means together with standard deviations for body measurements and blood data, with split according to the three study groups, for each measurement time: *Time 1*—before taking part in the walking regime, *Time 2*—after 7 weeks of walking, *Time 3*–7 weeks after walking regime cessation (*Time 1* + 14 weeks). No walking was required from our participants between measurement times *Times 2* and *3*.

	Control group—no walking (*N* = 27)			City walking group (*N* = 30)			Park walking group (*N* = 27)		
	*Time 1*	*Time 2*	*Time 3*	*Time 1*	*Time 2*	*Time 3*	*Time 1*	*Time 2*	*Time 3*
Weight (kg)	68.63 (13.45)	69.60 (12.99)	68.81 (13.81)	65.50 (13.99)	65.69 (13.99)	65.30 (13.77)	63.04 (15.89)	64.92 (15.54)	64.83 (16.61)
Height (m)	1.70 (0.09)			1.67 (0.08)			1.66 (0.08)		
BMI (kg/m^2^)	23.71 (4.47)	23.97 (4.30)	23.84 (4.47)	23.20 (3.94)	23.52 (4.20)	23.40 (4.14)	23.15 (5.11)	23.47 (4.85)	23.72 (5.67)
Cholesterol (mg/dL)	180.07 (33.89)	176.11 (31.16)	183.33 (34.68)	185.87 (47.69)	170.03 (38.84)	173.27 (44.45)	192.70 (30.56)	169.96 (36.79)	173.11 (47.82)

Note. For each of the monitored variables, no significant differences were recorded between the three study groups (control vs. park walking vs. city walking) for each of the *Time* 1–3 measurements taken (all *ps* > .09). Additionally, the control group, which had no walking requirement, exhibited no significant differences in each of the analysed characteristics, across the three measured times.

**TABLE 2:** Average daily recorded motricity (means ± SD) for the two experimental groups of city and park walking, as monitored on individual Mi 5 bands together with MiFit app, over a period of 7 weeks between January and March 2022.

	City walking group	Park walking group
Daily mean number of steps ± SD (count)	6,695.29 (1,379.68)	6,817.31 (2015.48)
Daily mean walked distance ± SD (m)	4,763.56 (991.24)	4,699.46 (1,421.32)
Daily mean consumed calories ± SD (kcal)	198.79 (70.80)	229.99 (117.76)

**TABLE 3:** Descriptives for the considered questionnaires total scores data.

	Control group-no walking			City walking group			Park walking group		
	Mean (SD)	Min.	Max.	Mean (SD)	Min.	Max.	Mean (SD)	Min.	Max.
Cardiovascular risk index (***CRI***)	0.67 (1.00)	0	4	0.60 (0.81)	0	3	0.52 (0.64)	0	2
Environment score (***ES***)	342.07 (57.65)	198	435	358.33 (50.20)	184	422	332.56 (77.76)	181	462
Fitness state (***FS***)	14.89 (4.72)	6	22	15.40 (3.45)	8	21	14.70 (3.96)	8	21

**FIGURE 3:**
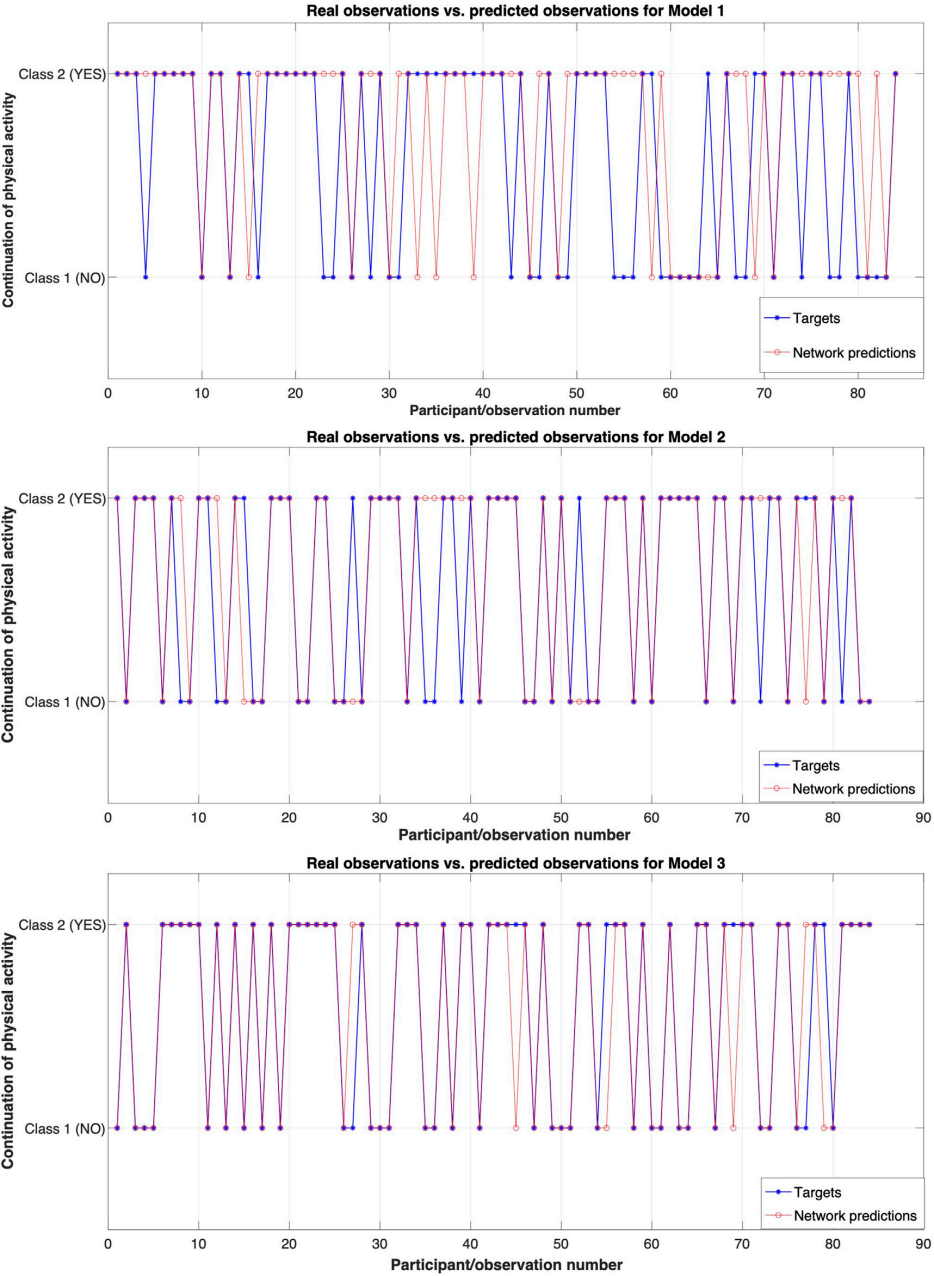
Real observations (targets) and predicted observations for the three analysed models. Model 1 includes four predictors, Model 2 includes 5 predictors, and Model 3 includes 6 predictors.

**FIGURE 4:**
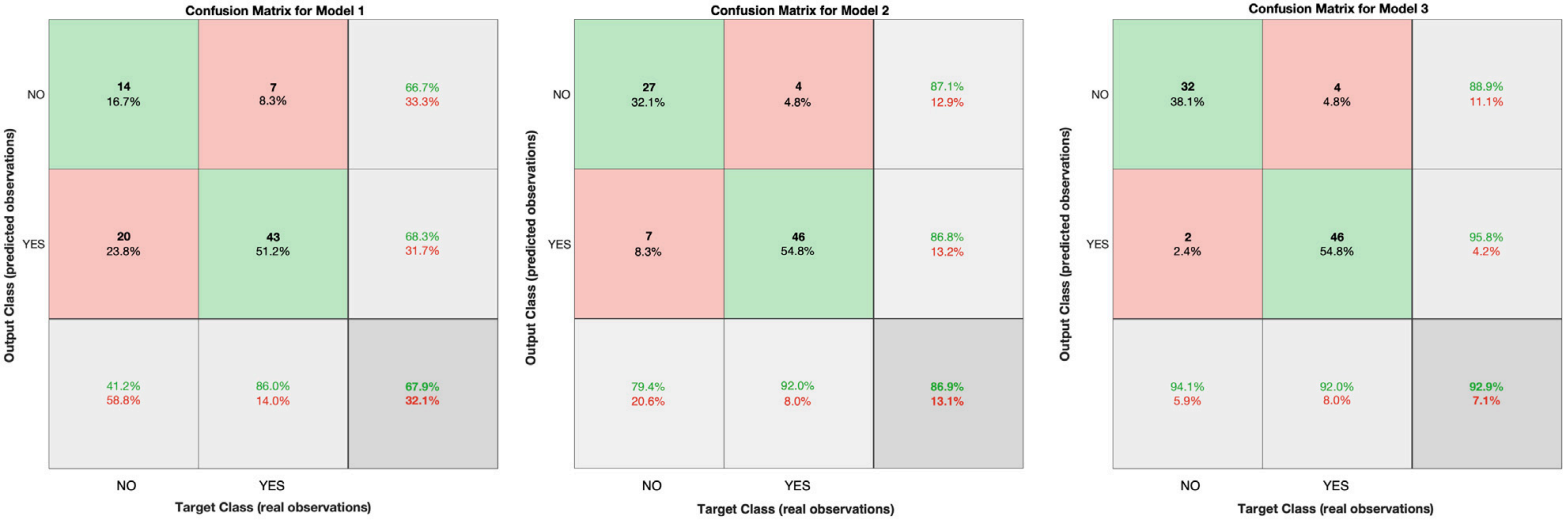
Confusion matrices for each of the three models conducted in this study.

**FIGURE 5:**
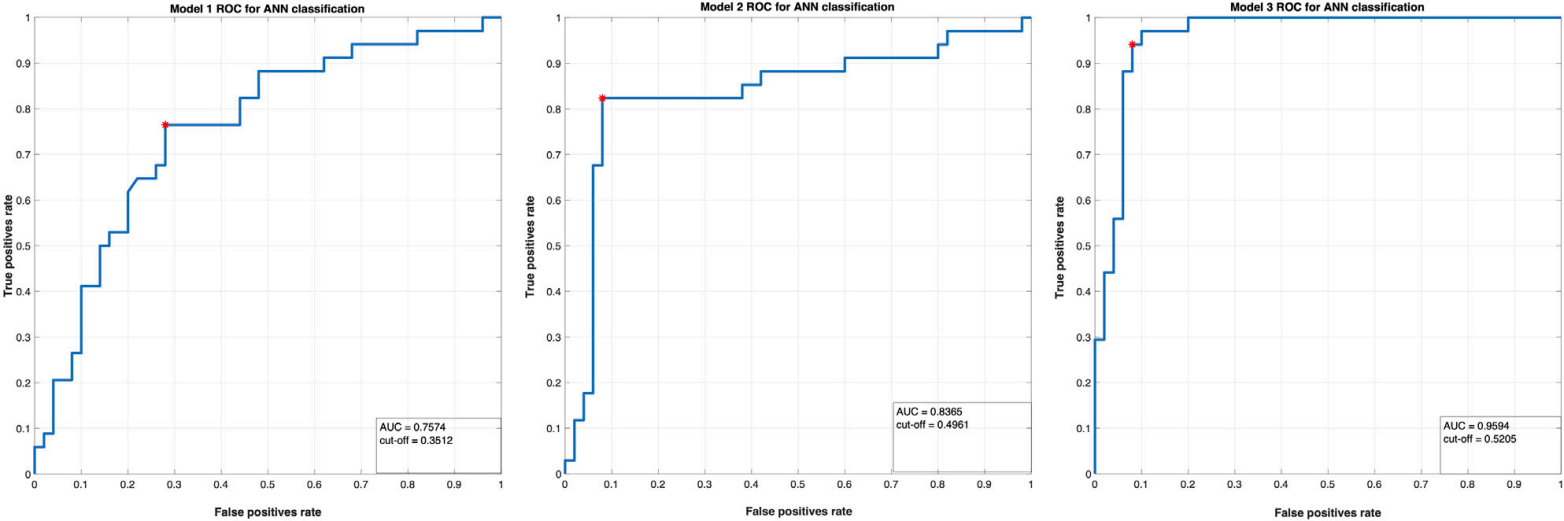
The ROC curves for the two classes in the three ANN models conducted in this study.

Figures 3–5 highlight the result and robustness of the ANN algorithm estimated in a hierarchical approach, i.e., Model 1 encompasses the variables of experimental group (***EG,*** control vs. city walking vs. park walking), the total score for the CVD risk questionnaire (***CRI***), the environment questionnaire (Spittaels et al., 2009, ***ES***), and the fitness state (Ortega et al., 2011, ***FS***), Model 2 also takes into consideration the ***BMI***, whereas Model 3 includes, in addition to all the variables above, the total blood cholesterol measured at study *Time 2* (***Ch***).

Figure 3 displays the observed classes (blue) and the predicted classes (red) by the model. The performance (error) for these models is: *MSE*_Model 1_ = 0.1972, *MSE*_Model 2_ = 0.1389, and *MSE*_Model 3_ = 0.0753. Given that when optimization is considered the *MSE* is typically subject to minimization, i.e., the lower value indicates a better fit of the estimates to actual values, these values highlight that Model 3 has the best accuracy or, in other words, a better fit. That is, adding blood cholesterol information to the self-reported questionnaire and BMI calculations is an enhanced predictor of continued/sustained physical activity.

See Figure 4 for the confusion matrices computed for the training set, to illustrate this enhanced prediction of sustained physical activity from the added bodily physiological data. A confusion matrix represents a table typically used to describe the performance of a classification model on a set of test data for which the true values are known. The green diagonal elements represent correctly classified observations, while the red diagonal elements represent misclassified observations. As it can be observed, a total of 67.9% of observations were correctly classified by Model 1, a total of 86.9% of observations were correctly classified by Model 2, and a total of 92.9% of observations were correctly classified by Model 3. As such, Figure 4 highlights that when we add new predictors related to the BMI (in Model 2) and total blood cholesterol (in Model 3) into the system, we can classify with significantly increased precision whether a person will continue the physical activity already commenced, or not. That is, adding physiological features to our first model based exclusively on questionnaire data, considerably enhances the overall model predictive performance.

Another well-established way to address the performance of a model is by plotting a Receiver Operating Curve (ROC). The ROC is a metric commonly utilised to verify the quality of classifiers and it is achieved by plotting the true positive rate (i.e., a Class 2—YES option correctly classified by the model as YES, sensitivity) against the false positive rate (or false alarms rate, i.e., a Class 1—NO option incorrectly predicted as a YES class, 1—specificity, as reflected in the number of false positives divided by the sum of the false positives and the true negatives, indicating how often a positive class is predicted when the actual outcome is negative) at various threshold settings; see Figure 5. Classifiers that give curves closer to the top-left corner of Figure 5 indicate a better performance for Model 3.

Lastly, the area under the ROC curve (AUC) is another valuable metric used to summarize the overall classification of a model’s performance. The AUC value indicates how able a model is to distinguish between designated classes. That is, the higher the AUC, the better the model is at predicting 1-labelled classes as 1 (NO) and 2-labelled classes as 2 (YES). The calculated AUC for Model 1 is AUC = .7574, indicating a fair discrimination capacity to distinguish between the two classes. The calculated AUC for Model 2 is AUC = .8365, with a good accuracy to distinguish between the two analysed classes. The calculated AUC for Model 3 is AUC = .9594, highlighting an excellent discrimination capacity to distinguish between the two monitored classes. In addition, the optimum cut-off point was defined as the point that maximized the AUC value (see also Perkins and Schisterman, 2006; Unal, 2017). These cut-off points (0.3512 for Model 1, 0.4961 for Model 2, 0.5205 for Model 3) provide support in discriminating between participants that have less physical activity as compared to the study start (Time 1) and those that continued the physical activity (as this was assessed at Time 3).

## Discussion

Walking contributes to health and is a promoting habit for daily living and quality of life. Here we explored those psychological and physiological contributors to maintaining a healthy physical activity habit, once this has commenced. For this, we monitored a group of participants who were required to walk through the city, to walk in the park, or to not walk at all, for a continuous period of 7 weeks. We registered questionnaire responses and health measurements, before the start of programme and after this was completed. Further, to investigate behavioural habit formation, we re-called participants in the lab after a period of 7 weeks following the end of the required walking regime, to perform further health measurements, as well as to interview them with regard to their physical activity continuation.

Highlighting no significant change in blood cholesterol for the experimental walking groups, our results are in agreement with other intervention studies that have failed to evoke favourable changes in lipid parameters (Albarrati et al., 2018; Buyukyazi et al., 2010; Kelley, Kelley and Tran, 2004; Murtagh et al., 2015; Murtagh, Boreham, Nevill, Hare and Murphy, 2005; e.g., a quantitative analysis of blood lipid adaptations to exercise revealed that the threshold for adaptation occurs at training volumes of 1,200–2,200 kcal/week). With the total energy cost of the walking sessions in the present study situated at the lower predicted training volumes, the lipid profiles may have not been altered consistently. The lack of body mass change is also in agreement with other walking studies using normal-weight participants (Kelley et al., 2004; Murtagh et al., 2005). During the present 7 weeks walking intervention, it is estimated that participants expended on average between 7,000–8,000 kcal while walking, and therefore, theoretically should have incurred a weight loss of approximately 1 kg (American College of Sports Medicine, 2013). A compensatory increase in food intake in response to increased energy expenditure during walking sessions, or a reduction in leisure-time activity has been proposed to account for such an effect (Murtagh et al., 2015). Previous studies have not described significant changes in total cholesterol and its subfractions with walking (Kelley et al., 2004; Albarrati et al., 2018). One possibility may be that not weeks, but rather months of endurance exercise involving a minimum expenditure of about 1,200 kcal per week are needed to better blood lipids in typical hyperlipidemic participants. Another explanatory path is that participants in both the city and park walking in our study took a not so endurance-based, but rather a mental immersive or attentive approach to physical activity, by actively experiencing the surrounding environment, a walking workout commonly referred to as *deep travel* (Hiss, 2017).

Further, mirroring previous findings on blood lipids, no significant differences were found in our study between walking in the park and city-walking. However, other longitudinal studies, like ours, indicate that participants do not discern between benefits offered by the urban and natural environments, highlighting that a *personal* stance is taken when a walking context is evaluated: Each participant is likely to develop a preference, and this is given by their own interaction with the surrounding environment (Marselle et al., 2013; Cooley et al., 2021). Note that, at least when walks were taken in the space of natural farms, a higher wellbeing could be predicted (Marselle et al., 2013). Hence, walking in isolation cannot be used to induce therapeutic changes in blood lipids, but its effects cannot be ignored on other health indicators like the general fitness level, maximum oxygen consumption, body composition, physical activity, and blood pressure (Kelley et al., 2004).

For this reason, an important distinction needs to be made with respect to green spaces located in nature, outside the city realm, and green spaces purposefully organised within the city space. In other words, how relevant is the distinction between city and park, when the park itself is located within the city perimeter? It has been demonstrated that even minimal contact with natural landscapes, such as admiring the view from one’s window, reduces stress (Ulrich, 1984; Elsadek et al., 2020). One explanation is that we might simply prefer natural landscapes to urban ones (Chang and Chen, 2005; Velarde et al., 2007; Meidenbauer et al., 2020), with a virtual simulation of the natural environment shown to contribute to an enhanced wellbeing state (Browning et al., 2020). On the other hand, bar the attractivity of the urban spaces’ lifestyle, facilities, and the opportunities they offer (Lecic-Tosevski, 2019), it has been argued that they represent a threat to mental health, by promoting isolation, with urban visual and auditory pollution enhancing anxiety and/or depression (Okkels, Kristiansen, Munk-Jørgensen and Sartorius, 2018; Klompmaker et al., 2019; Lan, Roberts, Kwan and Helbich, 2020; Li, Martino, Mansour and Bentley, 2022). In line with our findings highlighting that a person’s environment contributes to the continuation of an already started physical activity, research has shown that the urban scape needs quality relationships with neighbours, as it has been shown that city neighbourhood impacts health, with higher blood cortisol reported for violent environments (Do et al., 2011; Ettema and Schekkerman, 2016). In addition, it is a fact that people without access to parks and recreational services (i.e., a friendly environment for exercise) are less likely to engage in physical activity (Taylor et al., 2007).

Knowing that both physical activity and nature independently enhance human health, we expected measured health benefits derived from being active in green or natural places to be greater than the benefits of walking in the urban environment. Our results could be taken to indicate that physical exercise is crucial, whichever the environment (natural or urban) of practice. That is, *any* movement is beneficial movement. For example, researchers investigated a 50-min walk along a forest path, or along a busy road, and the same period of activities of daily living and found that physical activity improved psychological parameters (e.g., positive and negative affect, anxiety, perceived stress, and working memory), regardless of the walking atmosphere (Koselka et al., 2019). A moderating factor could be age, as demonstrated by a study on participants with an average age of 65 years: Roe et al. (2020) were able to highlight the positive effects of walking through a quiet urban area with front gardens and street trees, as opposed to moving through an urban busy *gray* district. Relatedly, in line with our present results, residing in a ‘green’ neighbourhood does promote walking (Inoue et al., 2010), prevents obesity and CVD, and importantly, it also appears to favour weight loss (Chandrabose et al., 2019).

It has been demonstrated that we need between 18 and 254 days to form a new behavioural habit, a process with high variability among participants and an average of 66 days needed for the tested new habits (e.g., running 15 min in the evening) to become automatic (Lally et al., 2010). Because of the implications for health, it is noteworthy to investigate long-term motor habit formation, especially taking into account that when we consider exercise, we tend to typically acknowledge the long-term benefits for our health and wellbeing. However, at the same time, we are likely to oversee any immediate benefits in physical exercise, and rather focus on its short-term costs and regard exercising as a time-/money-consuming effort (Rice, 2013). Further, the specific time span of targeted behaviour (i.e., the total duration monitored) needs to be experimentally assessed. For instance, behaviour change theory has long delimited several stages to intervene and change a given behaviour of interest, including the precontemplation, contemplation, preparation, action, and maintenance periods (Prochaska and DiClemente, 1983). The maintenance stage has been typically settled at 6 months for physical exercise behaviour. Note that in our design we have directly targeted the maintenance period, by opposing a behaviour-requested-period (e.g., 7 weeks of walking) to a subsequent no-requirement period, also amounting to 7 weeks.

To identify specific drivers of long-term physical activity habit formation, here we utilise cascading ANNs models and highlight several relevant predictors of one’s propensity to continue a physical activity programme, based on walking. We consider the construction of cascading models from multiple sources of monitored data to be the highlight of our study. Because the probability distribution and the prior probabilities of classes do not constitute a requirement, ANNs are widely used in the pattern classification processes. It has been suggested that the popularity of neural networks in pattern classification, as opposed to logistic regression, could be specifically due to the consideration of these algorithms as powerful nonlinear generalizations of logistic regression (Dreiseitl and Ohno-Machado, 2002). Our results successfully highlight a considerably low mean square error for the tested ANNs, indicating that the chosen algorithm is an excellent predictive tool. The current study ANN models first take into account the easily available questionnaire data, indicating that by only asking the appropriate questions from our participants, we can draw a baseline prediction of one’s propensity to continue an already started physical activity at 68% probability–That is, self-reported questionnaire data gives an overall good prediction of one’s continuation of an already started physical activity such as walking, based on participants’ self-assessed fitness level, their risk of CVD, other variables related to their living and work environment, as well as the city they live in, together with the type of environment where they performed the 7 weeks physical activity programme. Of all our models, we underline those variables related to body composition and total blood cholesterol level to significantly improve the classification performance: The relevant questionnaire data enhanced with physiological predictors (e.g., the BMI and total blood cholesterol levels) permit to successfully decide (i.e., with 93% probability) whether a novel participant will continue with the ongoing physical activity or not. To consider the practical value of our results, they indicate that a rightfully-selected self-report series of questionnaires are able to predict to a certain reasonable extent whether a given user of a potential (lifestyle) app would continue a given physical activity regime already started. Our data also highlight the type of physiological data needed to be considered for highly successful prediction of health outcomes.

With further consideration of potential limits of our study (e.g., total available physiological variables, the choice of specific physiological variables, sample size), for generalizability, a comprehensive assessment of bodily health could *ideally* sample from a broader range of physiological variables, including, amongst others, further body composition variables, blood pressure, blood sugar levels, inflammatory markers, and overall physical and mental wellbeing. Nevertheless, for the specific purpose of the present study (i.e., to predict the continuation of an already started sustained physical activity), the success of our prediction models is evident in the ANN metrics outlined in the Results section and highlights those specific relevant predictors for the continuation a sustained physical activity such as walking.

Taken together, with the well-established health benefits of physical exercise and nature exposure, here we successfully highlight *how* they both contribute to predict the continuation of an already started physical regime: With our ANNs, we can predict with excellent accuracy, whether a person commencing physical activity is going to successfully abide by it, or not. In this respect, our results are the first to evidence the contribution of specific factors to sustained physical activity, such as walking (i.e., either self-declared, as given by questionnaire data, or indicative of one’s health, as given by the BMI and measured total blood cholesterol). They allow to design future personalised engagement-motivated physical activity and/or general health early behavioural interventions for targeted populations of interest. By reliably identifying individuals who are likely to be (non)-compliant with a long-term physical activity regime, our evidence-based approach and algorithm has the potential to successfully enhance future interventions designed for the improvement of lifestyle-related physical activity. Considering the post-pandemic population focus on physical activity, health and physical-exercise-derived health, these results are of high importance, as they indicate a significantly bettered prediction of one’s success in maintaining an already started physical regime, when this is made based on physiological, established and easily available and economical health parameters.

## Data Availability

The raw data supporting the conclusion of this article will be made available by the authors, without undue reservation.
